# Harbour Porpoises *Phocoena phocoena* in the Eastern Scheldt: A Resident Stock or Trapped by a Storm Surge Barrier?

**DOI:** 10.1371/journal.pone.0056932

**Published:** 2013-03-06

**Authors:** Okka E. Jansen, Geert M. Aarts, Peter J. H. Reijnders

**Affiliations:** 1 IMARES, Wageningen UR, Department of Ecosystems, Den Burg, The Netherlands; 2 Wageningen University, Department of Aquatic Ecology & Waterquality Management, Wageningen, The Netherlands; Leibniz Center for Tropical Marine Ecology, Germany

## Abstract

Coastal protection measures are planned and executed worldwide to combat the effects of global warming and climate change, in particular the acceleration of sea level rise, higher storm surge flooding and extensive coastal inundation. The extent to which these defensive measures may impact coastal and estuarine ecosystems is still poorly understood. Since the building of a storm surge barrier, movement of harbour porpoises *Phocoena phocoena* in and out of the Eastern Scheldt tidal bay (SW-Netherlands) may be limited. To measure residency, porpoises stranded along the Dutch North Sea coast between 2006 and 2008 were sampled for muscle (n = 102) and bone tissue (n = 118), of which 9 muscle (8.8%) and 12 bone samples (10.2%) were collected from animals stranded within the Eastern Scheldt. Stable carbon (δ^13^C) was analysed to get insight into the habitat use and residency of porpoises in the Eastern Scheldt. Our data showed significantly higher δ^13^C values in the muscle of porpoises stranded within the Eastern Scheldt (µ = −17.7‰, SD = 0.4‰) compared to animals stranded along the Dutch coast (µ = −18.3‰, SD = 0.5‰). This suggests that most porpoises stranded in the Eastern Scheldt foraged there for a longer period. The distinct δ^13^C signature of animals from the Eastern Scheldt was not observed in bone tissue, suggesting a relatively recent shift in habitat use rather than life-long residency of porpoises within the Eastern Scheldt. The high number of strandings within the Eastern Scheldt suggests a higher mortality rate compared to the Dutch coastal zone. Our study indicates that along with other changes in the physical environment, the storm surge barrier may play an important role in determining the residency of porpoises in the Eastern Scheldt, and that the area might act as an ecological trap for porpoises entering it.

## Introduction

The predicted consequences of climate change and global warming on human populations and coastal ecosystems, in particular the accelerated sea level rise, higher storm surge flooding and extensive coastal inundation [1], [2], has led to increased coastal protection measures [3], [4]. These include the construction of seawalls, levees and flood gates, tidal barriers and beach nourishment [5]. For marine mammals, such defensive measures can lead to habitat loss, degradation and fragmentation. This can have important consequences for small populations due to chance effects and loss of genetic diversity [6].

For the Netherlands, where 60% of its land is situated below sea level [7], these defensive measures are particularly necessary. One such measure is located at the entrance of the Eastern Scheldt, a tidal bay, situated in the south-western part of the Netherlands. Two large auxiliary compartment dams were built between 1977 and 1987, isolating the former estuary from freshwater input of the river Scheldt and river Rhine. A storm surge barrier with gates was built between 1979 and 1986 at the entrance to the Eastern Scheldt to safeguard the tidal ecosystem while reducing the risk of flooding [8]. Comparable coastal protection structures are likely to increase world-wide in response to the effects of climate change [3], [5], and even semi-open structures may have detrimental effects on coastal and estuarine ecosystems.

Harbour porpoises *Phocoena phocoena* are the most common small cetaceans in Dutch coastal waters. Their abundance in Dutch coastal waters has changed significantly over the past decades. After a decline and near extinction in the 1950s and 1960s, numbers slowly recovered from the mid-1990s onwards, with a distinct peak in sightings and strandings in 2006 [9], [10]. Recent population estimates for the Dutch Continental Shelf (DCS) are approximately 26 000 in summer and 30 000 in autumn, with peak densities of up to 86 000 animals in March [11], [12].

Harbour porpoises are listed as endangered in several international, European and national legislations. They are also listed in several conventions, agreements and action plans such as the Habitats Directive (92/43/EEC), the Bern Convention, CITES and the ASCOBANS North Sea conservation plan under the Convention of Migratory Species) [10]. The conservation of species requires that we know enough about their ecology (e.g. migration, abundance, distribution, feeding ecology, reproduction, etc.) and their habitat in order to develop effective protection measures.

Anecdotal data shows that porpoises used to be common visitors in the Eastern Scheldt. However, for a few decades after the building of the storm surge barrier, no porpoises were observed in the area. Over the last ten years, small numbers of porpoises have been observed again in the Eastern Scheldt. They have become not only more abundant in the area but can now also be found there year round. Three dedicated annual surveys between 2009 and 2011 documented 37, 15 and 61 porpoises in the Eastern Scheldt, respectively, including 4–5 mother-calf pairs [13]. Currently it is unknown whether these individuals feed in the Eastern Scheldt for longer periods, or whether there is a continuous exchange between the Eastern Scheldt and the adjacent North Sea.

To analyse diet composition, trophic level and location in terrestrial and marine species, isotopic ratios of nitrogen (^15^N/^14^N, expressed as δ^15^N) and carbon (^13^C/^12^C, expressed as δ^13^C) can be used [14], [15]. Generally, predators are enriched in ^15^N compared to their prey (approximately 3–4‰ higher) [16], [17] while predator and prey are relatively similar in δ^13^C values (approximately 0.1–1‰ higher in predator) [17], [18]. δ^15^N values can therefore be used as indicators of relative trophic level [19] while geographic differences in δ^13^C can be used to indicate foraging location and habitat use of animals [20], [21]. Depending on the tissue analysed, stable isotopes reflect periods varying from hours to years [22], [23], [24]. Muscle tissue reflects assimilated diet of weeks or months [25] while bone tissue displays a more long-term integration, reflecting assimilated diet of several years [26], [27],[28]. Stable isotope analysis thus enables the identification of (longer-term) residency in areas with contrasting isotopic composition [29] such as the Dutch coastal zone *versus* the Eastern Scheldt.

A previous study based on stable isotope values of porpoises from Dutch coastal waters found differences in trophic level and feeding location between animals of different ages or sex, and identified different groups of porpoises that stranded during the summer and winter months. It was also found that the isotopic composition differed between animals stranded within the Eastern Scheldt compared to animals stranded along the Dutch coast [30].

The purpose of the present study is to use the existing and new analysis to gain additional insight into the habitat use and residency of harbour porpoises from the Eastern Scheldt by inspecting stable isotope composition of different tissues. To that end we have analysed a large number of porpoises stranded along the Dutch coast and porpoises stranded within the Eastern Scheldt and have assessed residency based on the isotopic composition (δ^15^N and δ^13^C) of muscle and bone.

## Materials and Methods

### Sample Collection

**Figure 1:**
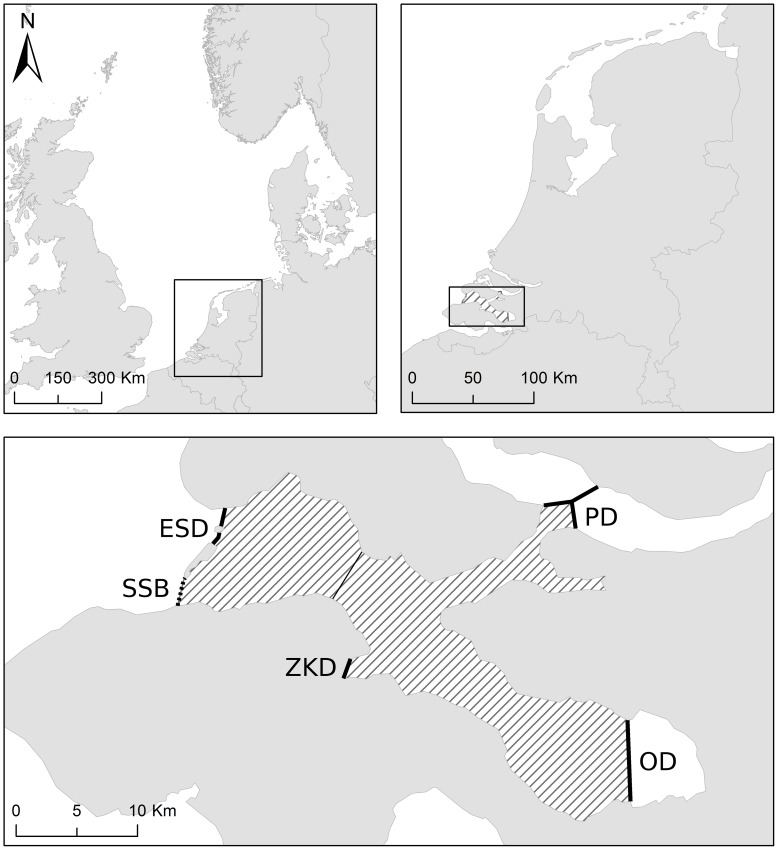
Eastern Scheldt tidal bay. The Eastern Scheldt tidal bay (dashed area), situated in the south-east of the Netherlands. Indicated are the Storm Surge Barrier (SSB), Eastern Scheldt Dam (ESD), Phillipsdam (PD), Oesterdam (OD) and Zandkreekdam (ZKD).

For this study, necropsy on 157 harbour porpoises, stranded on the Dutch coast and in the Eastern Scheldt (Figure 1) between 2006 and 2008, was carried out, resulting in 102 muscle and 118 bone samples. From 63 individuals, both bone and muscle samples were sampled. Dead porpoises were collected by staff and volunteers of the Dutch stranding network, coordinated by the NCB Naturalis in Leiden under the licence of the Dutch Ministry of Economic Affairs, Agriculture and Innovation (EL&I). Stranding date and location were reported for each animal, and during post-mortem examinations, general morphometric data were collected, *e.g.* sex (male, female, unknown) and length (cm). Muscle samples were taken from the ventral mid region, while for bone tissue, the 5^th^ rib was collected. Samples were stored frozen at −20°C until analysis. Stranding data of porpoises along the entire Dutch coast were taken from the database of the Netherlands Centre for Biodiversity (NCB) Naturalis [31].

### Sample Preparation

Sample preparation is described in detail in Jansen *et al*. [30]. In short, muscle samples were freeze-dried for approximately 20 hours, homogenized with a pestle and mortar and lipids were extracted using a 2∶1 chloroform-methanol solution. Bone samples (ribs) were cleaned and bone marrow was removed, they were sonicated in Milli-Q purified water and dried overnight. Bone samples were then homogenized with an automatic grinder, demineralized in a weak acid solution and dried overnight. Lipids were extracted from bone powder using a 2∶1 chloroform-methanol solution. All samples were analysed twofold: before pre-treatment to measure δ^15^N and δ^13^C values and after lipid extraction and acidification to measure δ^13^C values.

### Stable Isotope Analysis

Stable isotope measurements were performed by isotope-ratio mass spectrometry using an mass spectrometer (V.G. Optima Isoprime, UK) coupled to a N-C-S elemental analyser (Carlo Erba) for automated analyses at the Laboratory for Oceanology, Liège University, in Belgium. Stable isotope abundances are expressed in conventional delta (δ) notation in parts per thousand (‰), and are expressed relative to the international standards: Vienna-PeeDee Belimnite limestone (V-PDB) for ^13^C measurements and atmospheric nitrogen for ^15^N measurements. The following equation is used: δX = [(R_sample_ – R_standard_)/R_standard_] x 1000 where R_sample_ is the isotopic ratio of the sample and X is ^13^C or ^15^N and R the ratio of ^13^C/^12^C or ^15^N/^14^N [15], [20]. Reference materials used were: IAEA-N1 (δ^15^N: µ0.4‰, SD = 0.2‰) and IAEA-C6 (δ^13^C: µ = −10.4‰, SD = 0.2‰) (IAEA, Vienna, Austria). Internal standards (glycine) were inserted into all runs at regular intervals to calibrate the system and to assess drift over time. Measurement uncertainty, relative to true values of internal standards, was 0.1‰ for carbon and 0.3‰ for nitrogen. Further details can be found in Jansen *et al.* [30].

### Statistical Analysis

Homogeneity in variance was tested using the Bartlett test [32]. Generalized Linear Models (GLMs) [33] were fitted to examine whether variation in isotope values was associated with stranding location. Models were fitted using four possible response variables (i.e. bone δ^13^C (lipid extracted), muscle δ^13^C (lipid extracted), bone δ^15^N, muscle δ^15^N). The explanatory variable included stranding location (i.e. within the Eastern Scheldt or elsewhere along the Dutch coast). ANOVA F-tests were used to test if isotopic values differed between stranding locations. Statistical analysis was carried out in the computing environment R (R 2.92) [34].

## Results

### Porpoises

**Figure 2:**
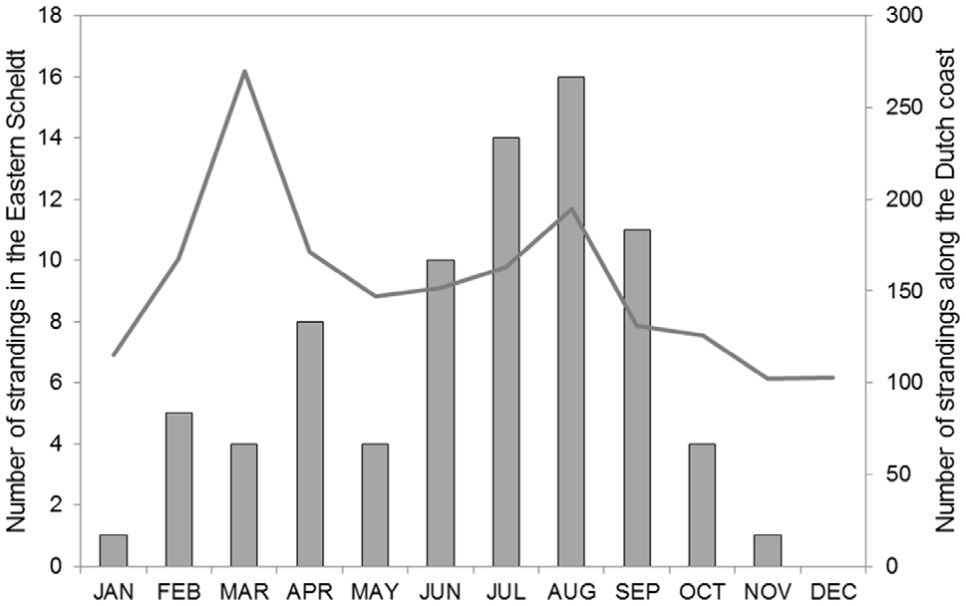
Porpoise strandings in the Eastern Scheldt. Porpoise *Phocoena phocoena* strandings in the Eastern Scheldt per calendar month (2006–2011). The solid line shows the number along the Dutch coast (www.walvisstrandingen.nl).

Between 2006 and 2008, 1189 stranded porpoises were documented along the Dutch coast and 37 within the Eastern Scheldt. The majority of bone and muscle samples were collected along the Dutch coast (n = 118 and 102, respectively). From the Eastern Scheldt, 12 bone samples (10.5%) and 9 muscle samples (8.8%) were available. Details on stranding location, sex ratio, age classes and length and weight measurements are presented in Jansen *et al*. [30]. From 2006 to 2008, the seasonal pattern in strandings along the Dutch coast reveals a peak in March and a slightly lower peak in August. In the Eastern Scheldt, most animals stranded in the months around August, and relatively few strandings were recorded around March (Figure 2).

### Isotopic Composition

**Figure 3:**
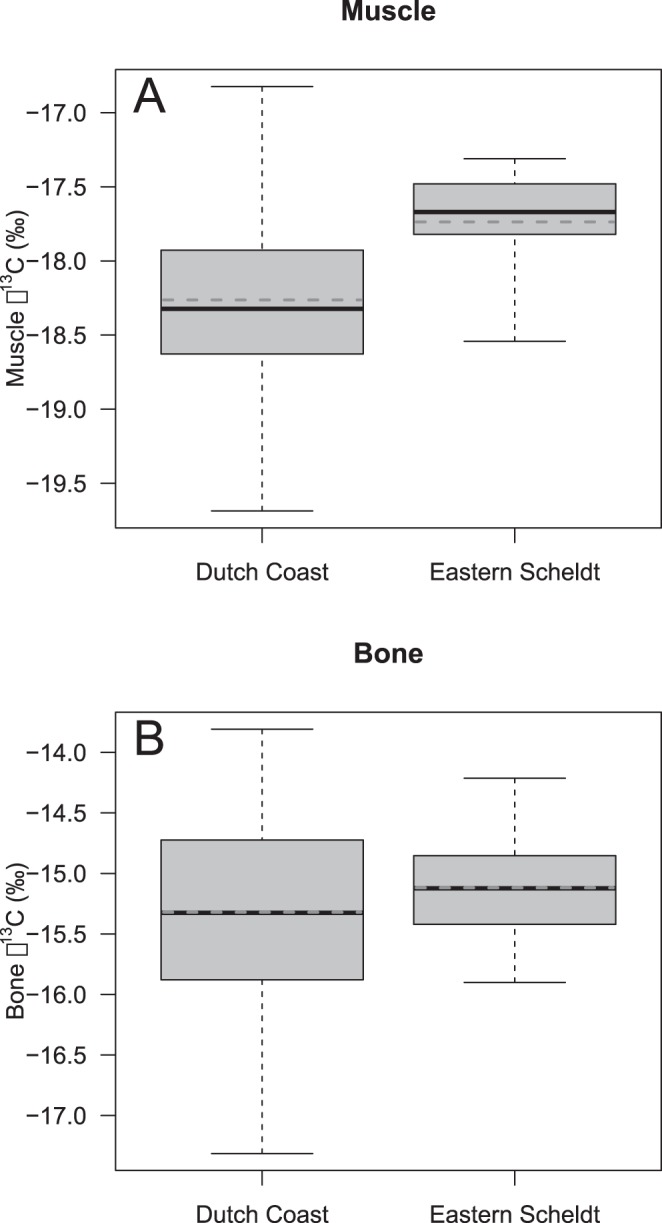
Porpoise δ^13^C values in muscle and bone tissue. Boxplot of δ^13^C values of porpoises *Phocoena phocoena* stranded within the Eastern Scheldt and elsewhere along the Dutch coast based on muscle tissue (A) and bone tissue (B). Solid line presents the median, dashed line represents the mean, the grey box represents the interquantile range (IQR, i.e. between 0.25 and 0.75 quantile) and the whiskers extend to the data extremes.

δ^13^C (lipid extracted) ranged from −19.7 to −16.8‰ (mean = −18.2‰, SD = 0.5‰) in muscle and from −17.3 to −13.8‰ (mean = −15.4‰, SD = 0.7‰) in bone (Figure 3). There was no evidence for heterogeneity in variance (muscle δ^13^C: Bartlett’s K^2^ = 1.13, p-value = 0.29; bone δ^13^C: K^2^ = 1.26, p-value = 0.26). GLMs revealed that the area of stranding (Eastern Scheldt *versus* Dutch coast) explained a significant (p-value<0.01) part of the variation of δ^13^C found in muscle tissue (ANOVA F = 8.993, p = 0.0034). The mean (and corresponding standard errors) for δ^13^C in muscle tissue from the Eastern Scheldt and Dutch coast were −17.74‰ (0.126‰) and −18.26‰ (0.053‰), respectively. In contrast, the δ^13^C values in bone tissue were −15.12‰ (0.163‰) and −15.32‰ (0.071‰), respectively, and there was no significant effect of stranding location (F = 0.81, p = 0.37).

δ^15^N ranged from 13.4 to 19.1‰ (mean = 16.4‰, SD = 1.4‰) in muscle tissue and from 11.3 to 20.7‰ (mean = 16.3‰, SD = 1.7‰) in bone. There was also no significant effect of stranding location on δ^15^N in muscle and bone (respectively, F = 3.6, p = 0.060 and F = 1.37, p = 0.25).

## Discussion

Since isotopic composition in a predator depends on tissue composition and lipid content [35], [36], tissue turnover-rates [22] and tissue-dependent fractionation [16], [18], we have taken into account the influence of these factors in assessing diet and feeding location of our study animals. The ability to infer irrefutable information on diet and feeding location of porpoises depends on the knowledge of the specific influence of these factors on porpoise isotopic composition [20].

Isotopic data from porpoises stranded along the French, Belgian and Dutch coastal waters [37] show similar δ^15^N values (µ = 16.2‰) but lower δ^13^C values (µ = −16.4‰) compared to our study, suggesting that porpoises from these adjacent areas generally feed on a similar trophic level, but that regional differences in δ^13^C baseline values are found in porpoises. Our data supports the findings of Christensen & Richardson [38] who have found gradually decreasing δ^15^N and δ^13^C values during the last century, possibly due to porpoises feeding on lower trophic level prey due to changes in the food web structure of the North Sea.

### The Eastern Scheldt

The Eastern Scheldt tidal bay was created by the building of compartment dams isolating the former estuary from freshwater input of the river Scheldt and river Rhine [8] (Figure 1). In addition, a storm surge barrier was built at the entrance of the Eastern Scheldt, reducing tidal amplitude in the bay. Since the existence of these constructions, changes in hydrodynamics (e.g. increased residency of the water mass), changes in hydrochemistry (e.g. increase of inorganic nutrients and particulate organic carbon) and related shifts in phytoplankton assemblage have occurred [8]. Though there are no baseline isotopic values available for the Eastern Scheldt, tidal estuaries are generally characterized by a long residence time of water and particles and a larger input of terrestrial organic matter. It can be expected that tidal estuarine systems differ significantly from other marine systems [39]. Clementz and Koch [40] have shown higher δ^13^C values in marine mammals from estuarine systems compared to those from offshore and near shore marine systems. Distinct (higher) δ^13^C values were found in muscle of porpoises stranded within the Eastern Scheldt compared to individuals stranded along the Dutch coast. This could either be caused by porpoises feeding on other prey species within the Eastern Scheldt, or by differences in baseline isotopic values of prey species of the Eastern Scheldt and the coastal waters. In any case, our data suggests that porpoises stranded in the Eastern Scheldt had fed in the area long enough to integrate the distinct isotopic pattern of this area and that they did not leave the area frequently to forage in the Dutch coastal zone. This distinct δ^13^C isotopic signature was not found in bone tissue, indicating a relatively recent shift in feeding location rather than life-long residency of porpoises within the Eastern Scheldt.

### Does the Eastern Scheldt Contain a Viable Population?

Three dedicated surveys of the Eastern Scheldt have documented between 15 and 61 porpoises, including 4–5 mother-calf pairs [13]. Most harbour porpoises along the Dutch coast are present in early spring, with a peak in March, and have departed by the end of April. Similar patterns are observed for the Western Scheldt and Wadden Sea [41]. This is most likely a seasonal migration as a result of prey availability. In contrast, in the Eastern Scheldt porpoises occur year round [13]. Furthermore, the seasonal pattern in strandings within the Eastern Scheldt is rather different from that observed along the North Sea coast. The dissimilarity between the sighting and stranding pattern suggest a seasonal disconnection between the Eastern Scheldt and other regions.

In 2011, 61 individuals were counted [42]. However, there is no detailed information on the processing of the data and hence the accuracy of that survey. But given the excellent weather conditions during the 2011 survey, the total count is thought to be a realistic estimate of the number of porpoises present in this area [13]. In that same year, 20 animals were found dead [30], suggesting that mortality is high and not sustainable if there would be no influx. It may be possible that some of these porpoises died in the North Sea and stranded in the Eastern Scheldt, but given the specific isotopic composition of porpoises stranded within the Eastern Scheldt, this is unlikely. Porpoises occurring in the adjoining North Sea are the likely source of this influx. Since the highest densities occur in March and April along the Dutch coast, most porpoises are expected to enter the Eastern Scheldt during that period. The fact that a specific isotopic signature in muscle tissue can be established in about 4–6 weeks [22], and that most strandings in the Eastern Scheldt occur between July and September, supports our conclusion that there must be a continuous influx of animals from the North Sea. The Eastern Scheldt may thus acts as an ecological trap for porpoises.

This study shows how porpoises may be affected by coastal protection structures and how even semi-open structures may form a barrier for migration and transit of these animals. This in turn influences their habitat use, residency and possibly also their survival. As the number of coastal protection structures is increasing worldwide, it is becoming more important to take into account the potential effect of such structures on marine organisms in order to meet and improve management objectives and conservation measures.

### Conclusions

Stable isotope analysis can provide important information on the feeding ecology and habitat use of harbour porpoises. Differences in isotopic composition were found between animals stranded along the Dutch coast and animals stranded within the Eastern Scheldt. We have shown that muscle δ^13^C values can be used to identify porpoises that have been feeding in the Eastern Scheldt for an extended period of time. Based on bone δ^13^C values, we have found no evidence that any of the animals analysed were born in the Eastern Scheldt, indicating that they have subsequently entered the area. Future stable isotope analysis of bone has the potential to assess whether animals born in the Eastern Scheldt stay there.

Mortality is exceptionally high in the Eastern Scheldt and both sighting rate and strandings differ seasonally compared to animals in the Dutch coastal zone. The increase in live animals counted in the past three years cannot be explained by calf production alone and observed relative mortality. Evidently, regular influx of animals from the North Sea must occur. Our study indicates that along with other changes in the physical environment, the storm surge barrier may play an important role in determining the residency of porpoises in the Eastern Scheldt. Additionally, in view of the relative high percentage of animals found dead, the area may act as an ecological sink for some of those immigrants.

Worldwide, coastal protection measures increase in response to the effects of global warming and climate change. The effects of these defensive measures on marine and estuarine ecosystems are still poorly understood. This study is an example of the impact of such a protection structure and highlights that even semi-open structures, which are meant to ameliorate habitat loss, degradation and fragmentation, may still affect the abundance and distribution of individual species.
